# Newly diagnosed AIDS patient with cerebellar JC virus

**DOI:** 10.1016/j.idcr.2023.e01842

**Published:** 2023-07-05

**Authors:** Sergio Alvarez-Mulett, Eli Sepkowitz, Douglas Sepkowitz

**Affiliations:** a Department of Medicine, NYP-Brooklyn Methodist Hospital, Brooklyn, NY, USA; b Department of Physical Medicine & Rehabilitation, Northwell Hospital, Manhasset, NY, USA; c Department of Infectious Disease, NYP-Brooklyn Methodist Hospital, Brooklyn, NY, USA

**Keywords:** JC virus, Cerebellum, Immune reconstitution

## Abstract

We present a case of a 57-year-old man with newly diagnosed acquired immunodeficiency syndrome (AIDS) infection who initially sought care for progressive dysarthria and gait instability. Neuroimaging and CSF studies revealed a diagnosis of progressive multifocal leukoencephalopathy (PML). Although the patient’s human immunodeficiency virus (HIV) decreased considerably in response to anti-retroviral therapy, he continued to deteriorate clinically. Ultimately, the central nervous system (CNS) lesions, which were once centered in the cerebellum, became expansile throughout his posterior fossa. There are few reported cases of cerebellar PML in patients with AIDS.

## Introduction

PML is a CNS demyelinating disease caused by the John Cunningham (JC) virus, which is a double-stranded DNA virus also known as Polyomavirus 2. JC virus infection is usually lifelong and asymptomatic. However, it may become pathogenic in immunodeficient patients. The IgG seroprevalence for JC virus has been calculated around 58% and it may increase with age [1]. At the time it was first described by Padgett in 1971, the number of cases was modest and mostly linked to lymphoproliferative disorders [2], [3]. However, the cases of symptomatic JC virus-related PML increased significantly in the 1980s and 1990s related to the exponential growth of HIV epidemic [4]. More recently, JC virus infection has been described in patients receiving anti-CD20 therapy and selective adhesion molecule (SAM) inhibitors [5], important medications in management of multiple sclerosis and other chronic inflammatory conditions.

## Case presentation

The patient presented to an outside hospital in March of 2022 with gait disturbances. He was found to have HIV, with a viral RNA of 315,000 copies/mL, and an MRI that revealed T2 hyperintense lesions within the left cerebellum and peduncle. He declined antiretroviral therapy, left against medical advice, and presented to our hospital the following week.

**Fig. 1:**
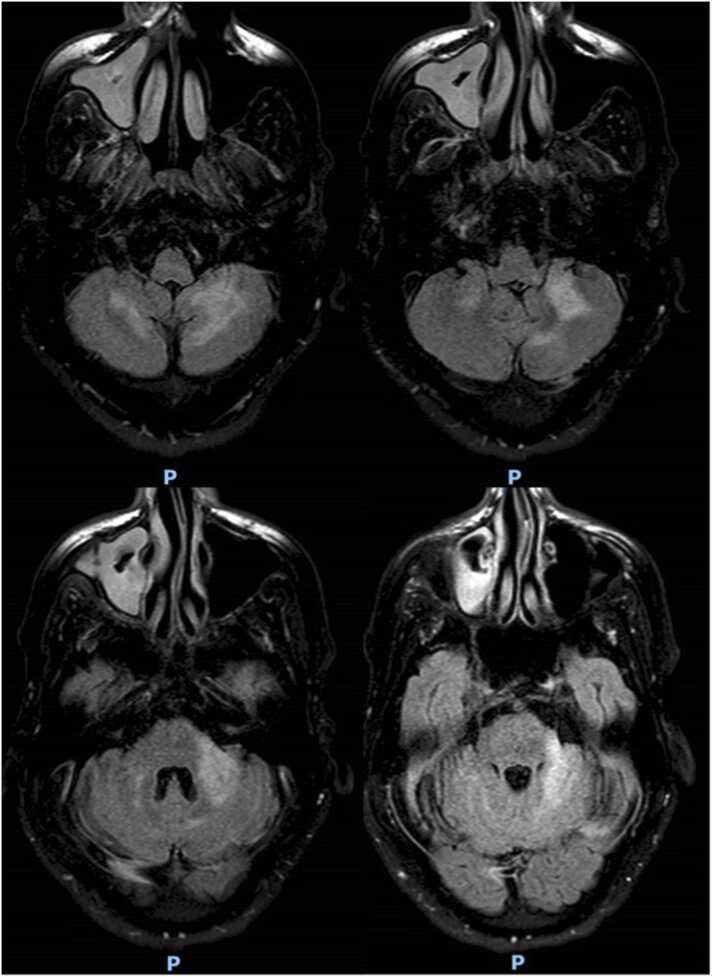
MRI Brain with and without contrast; axial T2 FLAIR (March 23, 2022). Subcortical T2 hyperintense signal abnormality involving the left brachium pontis/left cerebellar hemisphere with possible minimal right cerebellar involvement.

He was then started on bictegravir/emtricitabine/tenofovir alafenamide and a lumbar puncture confirmed the diagnosis of neuroinvasive JC virus, with a CSF DNA test of 2000 copies/mL (Table 1). At our hospital, the MRI of the brain revealed T2 hyperintense signal abnormalities in both subcortical cerebellar hemispheres, more pronounced on the left (Fig. 1).

**Table 1:** Select Laboratory Values.

Variables	March 2022	April 2022	May 2022
**Blood tests**			
WBC count min-max (x10^3/uL)	1400–3100	1800–26,290	1940–5740
CD-4 (cells/uL)	106	99	61
CD8 (cells/uL)	NA	312	568
CD4/CD8 Ratio	NA	0.32	0.11
HIV NAAT (copies/mL)	315,000	190	30
JCV by PCR	Not Detected	NA	NA
**CSF characteristics**			
Protein (mg/dL)	48	37	NA
Glucose (mg/dL)	63	76	NA
Red blood cells (cells/uL)	500	1	NA
White blood cells (cells/uL)	12	0	NA
Lymphocytes (%)	10		
Neutrophils (%)	2		
Cytology (malignancy screening)	Negative	Negative	NA
**CSF microbiology**			
JCV RT-PCR (copies/mL)	2000	14,000	NA
BKV PCR (copies/mL)	1990	Not Detected	NA

**Fig. 2:**
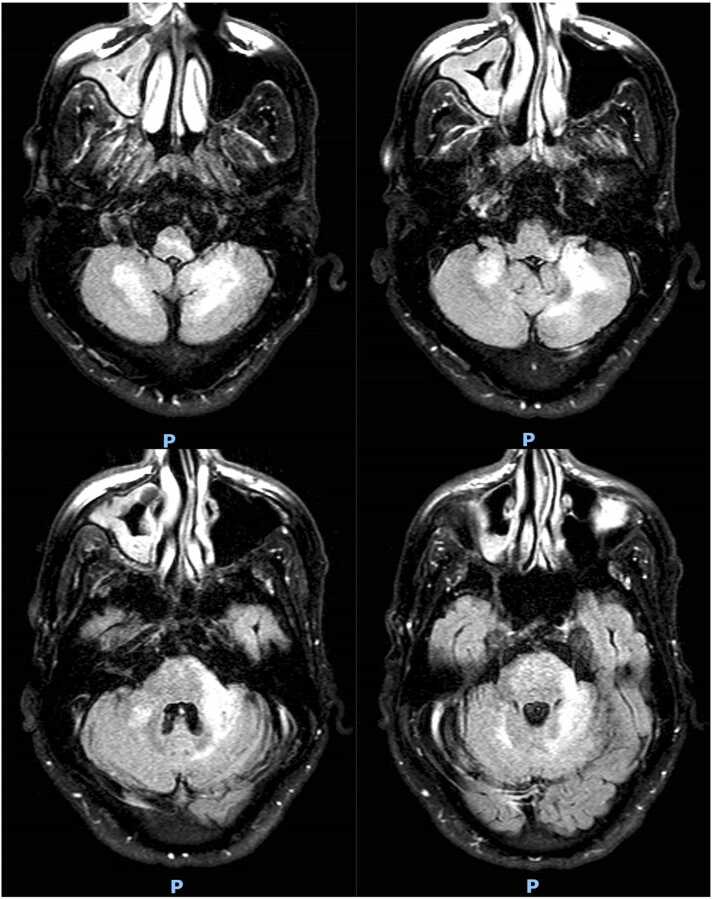
MRI Brain with and without contrast; axial T2 FLAIR (April 7, 2022). Stable geographical signal abnormalities of the posterior fossa compatible with the clinical diagnosis of PML. Stable additional nonspecific multifocal, overall mild signal changes of the supratentorial white matter.

**Fig. 3:**
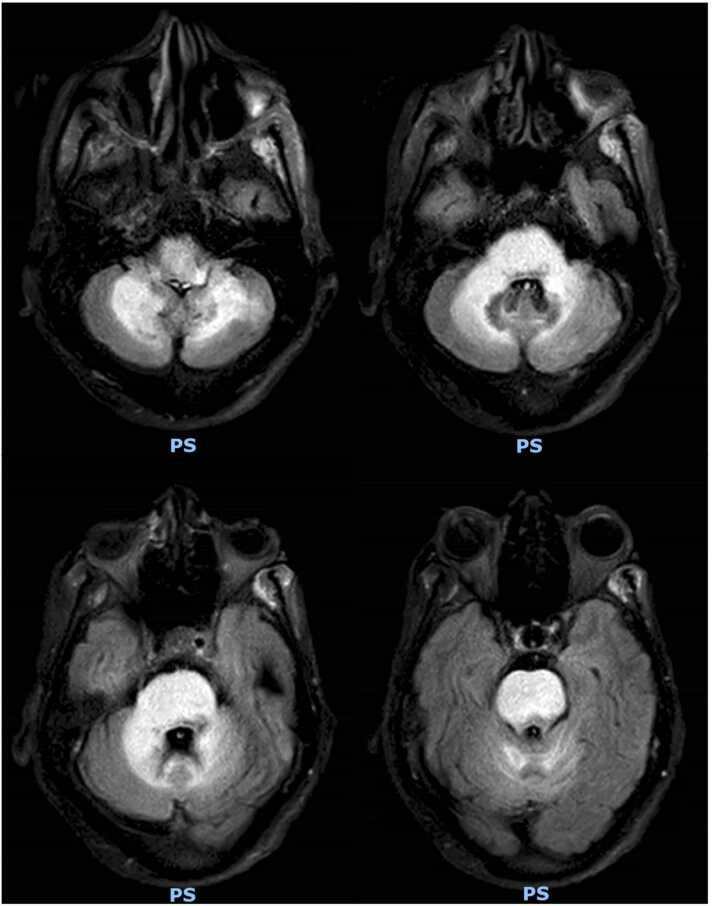
MRI Brain with and without contrast; axial T2 FLAIR (May 28th, 2022). Significant interval worsening of signal abnormalities in the posterior fossa with now diffuse involvement of the pons, extension to the mesencephalon and markedly more pronounced involvement of the medulla, right middle cerebellar peduncle, and central cerebellum. Patchy areas of restricted diffusion are without a clear vascular territorial distribution. IRIS is less likely in the absence of contrast enhancement, but it cannot be completely excluded in the appropriate scenario.

The patient was discharged without any changes in his neurologic symptoms. However, he returned the following month with a worsening gait disturbance, and new symptoms including dysarthria, dysmetria and left-sided nystagmus, despite a more than 3-log drop in his HIV RNA after 3 weeks of HAART (Table 1, Fig. 2). The rapid decrease of the HIV RNA raised concern for a possible immune reconstitution inflammatory syndrome (IRIS) and he was treated with systemic corticosteroids. Shortly thereafter, a second lumbar puncture revealed that the JC virus DNA had increased to 14,000 copies/mL. Over the remainder of his hospitalization the patient demonstrated improvements in his presenting symptoms and was discharged to an acute rehabilitation facility. After several weeks in the rehabilitation facility, he developed new neurological deficits including altered mental status, dysphagia, aphasia, worsening dysarthria and more pronounced left-sided hemiparesis. He was re-hospitalized and treated with pembrolizumab (2 mg/kg, single dose) but continued to worsen neurologically and a new MRI showed disease progression in the posterior fossa (Fig. 3). Ultimately, his family opted to pursue palliative measures, and he was discharged from the hospital to a hospice facility.

## Discussion

The incidence of symptomatic PML infection increased significantly in the 1990s, as the AIDS epidemic expanded. Later, the disease was observed in those receiving novel immunosuppressive therapies for chronic inflammatory conditions [5], [6].

The virus is thought to enter the body by inhalation or ingestion and can establish chronic infection in different tissues through its two forms: the archetypical (the transmissible), and the prototypical (the neuroinfective) form [7]. Within the CNS, the JC virus exhibits variable cellular trophism. For instance, characteristic demyelinating white matter lesions of PML are facilitated by positive feedback between antigens expressed by mature viral particles and 5HT2 receptors. Most commonly these are found in frontal and parieto-occipital lobes, and less frequently in the basal ganglia and posterior fossa [8].

Our patient had two unusual features: first, he presented with lesions initially confined to the cerebellum and its reciprocal tracks. Second, his clinical course was not ameliorated following antiviral medication-induced control of his HIV infection.

In cerebellar PML, the JC virus primarily targets granule cell neurons located in gray matter, which can lead to granule cell neuronopathy (GCN) [9], [10]. This separate entity seems to be related to a mutation in the major capsid protein VP1 protein [11], [12]. Indeed, when GCN occurs concomitantly with PML [13], cerebellar dysfunction is typical [14], [15], as observed in our patient. We suspect that his clinical presentation was mostly mediated by JC virus replication; however, we considered IRIS as a differential diagnosis.

IRIS associated to PML has been well described within the HIV population. His clinical worsening despite the control of his HIV infection was suggestive of IRIS [16], but the increase of the JC virus DNA in the CSF increased despite suppression of the HIV was more consistent with PML progression.

When treating IRIS, rapid initiation of systemic steroids has demonstrated benefit in several co-infections [16], [17]. However, the current literature in JC virus-related IRIS management is inconclusive [18], [19]. Thus, when the patient returned in the second month of his illness, he was treated with steroids for presumed IRIS without improvement. As a last resort, the patient received a novel PD-L1 inhibitor which has shown limited evidence for PML management by decreasing JC virus DNA due to a enhanced cellular immune response [20]. Unfortunately, by the third month, his lesions expanded throughout the posterior fossa, including the cerebellar cortex, coinciding with an increase in intrathecal viral DNA and his rapid clinical decline. To our knowledge, this sequence of events and its final outcome is unusual and have seldom been reported in the literature.

In summary, our patient with newly diagnosed HIV and cerebellar JC virus infection worsened dramatically after introduction of effective antiviral treatment. We hypothesize that this may be related to the distinct pathophysiology of JC virus in the cerebellum as well as the deleterious effects of immune reconstitution.

## Funding

None to declare.

## Ethical approval

N/A-case report.

## Consent

Written informed consent was obtained from the patient’s brother for publication of the details of their medical case and any accompanying images. A copy of the written consent is available for review by the Editor-in-Chief of this journal on request.

## CRediT authorship contribution statement

**Sergio Alvarez-Mulett:** Involved in clinical care, data analyses and interpretation, drafting the manuscript and editing various drafts for critically important intellectual content, final approval of version to be published, agreement to be accountable for all aspects of work related to the accuracy or integrity for any and all aspects of the work and its review. **Eli Sepkowitz**: Involved in clinical care, data analyses and interpretation, drafting the manuscript and editing various drafts for critically important intellectual content, final approval of version to be published, agreement to be accountable for all aspects of work related to the accuracy or integrity for any and all aspects of the work and its review. **Douglas Sepkowitz:** Involved in clinical care, data analyses and interpretation, drafting the manuscript and editing various drafts for critically important intellectual content, final approval of version to be published, agreement to be accountable for all aspects of work related to the accuracy or integrity for any and all aspects of the work and its review.

## Declaration of Competing Interest

The authors have no conflicts of interest to declare. All authors contributed to the article and agreed upon the final document. No ongoing research, sponsorship or funding pertains to the present report.
